# Changes in bowel sounds of inpatients undergoing general anesthesia

**DOI:** 10.1186/s12938-020-00805-z

**Published:** 2020-07-30

**Authors:** Guojing Wang, Mingjun Wang, Hongyun Liu, Suping Zhao, Lu Liu, Weidong Wang

**Affiliations:** 1 Key Laboratory of Biomedical Engineering and Translational Medicine, Ministry of Industry and Information Technology, Chinese PLA General Hospital, Beijing, China; 2 Department of Medical Engineering, Medical Care Center, Chinese PLA General Hospital, Beijing, China; 3 Bioengineering Research Center, Medical Innovation Research Division, Chinese PLA General Hospital, Beijing, China; 4 Anesthesia and Operation Center, Chinese PLA General Hospital, Beijing, China; 5 College of Otolaryngology Head and Neck Surgery, Chinese PLA General Hospital, Beijing, China

**Keywords:** Bowel sounds, General anesthesia, Intestinal function

## Abstract

**Background:**

General anesthesia can affect intestinal function, but there is no objective, practical and effective indicator to evaluate the inhibition and recovery of intestinal function. The main objectives of this study were to assess whether bowel sounds (BSs) changed before, immediately after and 3 h after general anesthesia, and whether these changes in BSs are an effective indicator of intestinal function and an accurate guide for postoperative feeding.

**Methods:**

We randomly selected 26 inpatients and collected three sets of 5-min continuous BS data before the operation (Pre-op), immediately after the operation (Pro-op) and 3 h after the operation (3 h-Pro-op) for each patient. Then, the linear and nonlinear characteristic values (CVs) of each effective bowel sound were extracted and paired *t* tests and *rank*-*sum* tests were used to evaluate the changes in the BSs.

**Results:**

The differences in CVs, between Pre-op and Pro-op, as well as between Pro-op and 3 h-Pro-op, were statistically significant (*p* < 0.05). However, there are no statistically significant differences between all the CVs between Pre-op and 3 h-Pro-op (*p* > 0.05).

**Conclusion:**

BSs change before and after general anesthesia. Furthermore, the BSs are weakened due to general anesthesia and recover to the pre-op state 3 h later. Therefore, the BSs can be an indicator of intestinal function under general anesthesia, so as to provide guidance for postoperative feeding, which is of considerable clinical significance.

## Background

General anesthesia can inhibit gastrointestinal function, so postoperative feeding needs to wait for the gradual recovery of gastrointestinal function to allow appropriate and timely feeding. At present, there is no objective or practical evaluation method for postoperative recovery of gastrointestinal function, so the time for resuming oral input is somewhat arbitrary. However, for patients undergoing general anesthesia, it is important to be able to take in nutrition relatively early for optimal postoperative recovery. Therefore, the evaluation of gastrointestinal function after general anesthesia directly affects the judgment of timely postoperative feeding time, which has important clinical significance.

Studies of noninvasive methods to judge the recovery of gastrointestinal function after general anesthesia mainly use anal exhaust [1], electrogastrogram [2], bowel sounds (BSs) auscultation and dynamic magnetic resonance imaging (DMR) [3].

Anal exhaust mainly depends on the patients’ subjective complaints, which cannot objectively and in a timely way reflect the recovery of gastrointestinal function. Electrogastrograms are more objective and accurate than anal exhaust assessment, but are easily influenced by other bioelectric signals, and the current analysis of electrogastrogram data is not particularly mature or widely accepted. DMR relies on large imaging equipment, so it is difficult to monitor patients in a timely way during the perioperative period.

Auscultation of BSs is an important noninvasive way to judge gastrointestinal function. BSs are produced by the movement of substances in the intestine, so the sounds can objectively reflect the activity of intestinal peristalsis in real time. Research on BSs use the characteristics of BSs to observe gastrointestinal status and diagnose gastrointestinal diseases. In clinical practice, the observation of gastrointestinal peristalsis is used to monitor feeding events, thus for example, providing a reference for the monitoring of blood glucose in an artificial pancreas system [4]; BSs can also be used as one of the indicative parameters of gastrointestinal diseases [5]. If the gastrointestinal tract development lesions occur, such as gastroduodenal disease, intestinal disease, and large bowel disease, the corresponding intensity or number of BSs may also be abnormal. In addition, BSs can indicate other diseases. Recent studies have found that BSs can not only indicate gastrointestinal state, but also have clinical significance for sepsis [6], Parkinson’s disease [7] and other diseases. The above studies show that BSs can reflect gastrointestinal function, so we can consider the application of changes in BSs in the evaluation of gastrointestinal function recovery in patients after general anesthesia.

However, using a handheld stethoscope for auscultation is still the main way to quickly obtain intestinal sounds in the clinic. As for the identifying the specific gut sounds, the results tend to be random and depend on of subjective judgment and are therefore questionable [8, 9]. In the study of BSs, the sounds are acquired by means of an assembly of mature pickups and storage units. Currently, there is no special bowel sound equipment in the clinical environment to collect bowel sound data.

In this study, considering the requirements of the perioperative medical environment and the patient’s poor cooperation before and after the operation, we used a self-developed wearable bowel sound recording device. The device can be easily attached to the patient’s abdomen, and the sound data can be collected and stored conveniently with no interference with the patients’ treatment.

The hypothesis to be tested is that the results of BSs analysis indicate the occurrence of changes in BSs in patients undergoing general anesthesia using our sound recording device, and that it provides theoretical and practical support for the use of bowel sounds as a reference index for post-anesthesia bowel function recovery evaluation and postoperative feeding.

## Results

The research design was approved by the Medical Ethics Committee of the Chinese PLA General Hospital for clinical research (No. 2018-176-01). We randomly selected 26 out of 339 inpatients from August to October 2019 in the Second Department of Otolaryngology, Head and Neck Surgery, at the Chinese PLA General Hospital. Each subject signed an informed consent form. We recorded clinical factors that might influence bowel sounds, including age, gender, BMI, and anesthetic type. The patients were also asked to confirm the absence of intestinal disease to eliminate abnormal changes in bowel sounds caused by gastrointestinal dysfunction. Three sets of 5-min continuous BSs were collected from each patient. The first set of data was collected before the operation (Pre-op) which was defined as the time after fasting for 24 h and before entering the operating room. The second set was collected after entering the recovery room and completing tracheal extubation (Pro-op). The last set was collected at 3 h after extubation (3 h-Pro-op) in the ward if conditions permitted. The acquisition location of bowel sounds was determined as the right lower abdominal region [10]. To minimize the influence of different devices and different operators on the accuracy of the tests, one person used the same device to test the subjects’ BSs during the experiment.

We obtained 70 sets of 5-min BSs from 26 patients as shown in Table 1. The 70 datasets consisted of 26 sets at preoperative, 26 sets at postoperative and 18 sets at 3 h after surgery.

**Table 1:** Patient data

Age (years)	39 (9–77)^a^
Sex (M/F)	15/11
BMI	24.88 (4.56)^b^
Operation time (min)	123.65 (67.06)^b^
Anesthesia type	General anesthesia

*BMI* body mass index

^a^Values are presented as mean (range)

^b^Values are presented as mean (standard deviation)

Characteristic values (CVs) were calculated for each effective bowel sounds (EBS), including 7 linear parameters and 8 nonlinear parameters. After statistical analysis, all the *p* values were adjusted using the false discovery rate (FDR) method, and a value of *p* < 0.05 was considered to indicate statistical significance; otherwise, no statistical difference was considered.

Table 2 shows the statistical analysis results of the CVs of EBSs between Pre-op and after Pro-op. The results show that in the linear time-domain parameter analysis of BSs, the frequency of EBSs, namely Num_bs, the number of BSs within 5 min, is statistically significant (*p *< 0.05). The parameters that can represent the intestinal sound energy include Mean_Mag_bs, Std_Mag_bs, and Sum_bs. Among these three parameters, Mean_Mag_bs and Std_Mag_bs had no statistical significance (*p *> 0.05), but there was a statistical difference (*p *< 0.05) in Sum_bs. The duration parameters of intestinal sounds included Mean_Duration, Std_Duration, and Sum_Duration, in which the difference between the two parameters of Mean_Duration and Std_Duration was not statistically significant (*p *> 0.05), while the Sum_Duration difference was statistically significant (*p *< 0.05). This indicated that the linear CVs that reflect occurrence frequency, overall energy and overall duration of EBSs and the nonlinear CVs that reflect the dispersion degree of stability and complexity of EBSs were statistically significant (*p *< 0.05). However, there were no statistically significant differences in the CVs reflecting the energy and duration, as well as the stability and complexity of local EBSs (*p *> 0.05). Therefore, the frequency of occurrence, the energy and the duration of BSs were affected by the operation general anesthesia and were weakened as a whole, but the energy and duration of local EBSs were not affected, indicating that general anesthesia affected the overall intestinal peristalsis intensity, but did not inhibit the local intestinal peristalsis state. In the nonlinear recursive parameter analysis, the mean values of RR, Lmean, ENTR and TT were not statistically different (*p *> 0.05). However, the standard deviation of RR, Lmean, ENTR and TT showed statistical differences (*p *< 0.05). RR, Lmean and TT all reflect stability of the signal, while ENTR reflects the complexity. There was no statistical difference (*p *> 0.05) in the mean of the recursive parameters of BSs, but there were statistical differences (*p *< 0.05) in the standard deviation of these recursive parameters, indicating that the dispersion degree of stability and complexity of the system became smaller.

**Table 2:** Statistical analysis results between Pre-op and Pro-op

CVs	Pre-op (*n* = 26)	Pro-op (*n* = 26)	*p* values
Num_bs	23.23 (13.61)	10.34 (10.86)	0.001*
Sum_bs	5,768,536 (4,326,873)	2,121,638 (1,928,966)	0.004*
Sum_Duration_bs	98,292 (52,350)	45,808 (41,512)	0.001*
Mean_Duration	0.500 (0.287)	0.515 (0.433)	0.790**
Std_Duration	0.660 (0.580)	0.411 (0.372)	0.108*
Mean_Mag_bs	58.177 (38.967)	47.921 (38.363)	0.494*
Std_Mag_bs	39.340 (32.061)	10.124 (35.826)	0.144**
Mean_RR	0.108 (0.050)	0.082 (0.028)	0.086**
Std_RR-	0.066 (0.064)	0.019 (0.034)	0.009**
Mean_Lmean	29.789 (6.750)	27.352 (12.817)	0.518*
Std_Lmean	12.089 (13.025)	6.564 (6.134)	0.017**
Mean_ENTR	4.030 (0.205)	3.947 (0.322)	0.518**
Std_ENTR	0.380 (0.105)	0.234 (0.167)	0.001*
Mean_TT	32.858 (8.974)	30.141 (16.471)	0.518*
Std_TT	17.147 (18.951)	8.405 (9.488)	0.010**

*Pre-op* before operation, *Pro-op* after operation

* Values of this line are presented as mean (standard deviation), and the statistical method is the *paired t test* because the data are normally distributed

** Values of this line are presented as median (quartile range), and the statistical method is the *rank*-*sum* test because the data are not normally distributed. CVs, characteristic values

Table 3 shows the statistical analysis of BSs between Pro-op and 3 h-Pro-op. The frequency of EBSs increased with statistical significance (*p *< 0.05) 3 h later. Compared with the Pre-op and Pro-op comparisons, the Mean_Mag_bs, Std_Mag_bs, Mean_Duration, and Std_Duration, which represent the mean and standard deviation of single EBS energy and duration, the differences were not statistically significant (*p *> 0.05). However, there were statistically significant differences (*p *< 0.05) between Sum_bs representing total energy and Sum_Duration representing total duration, both of which were larger, indicating that the overall energy and duration of BSs had recovered to a certain extent after 3 h. It was consistent with the statistical results of Pre-op and Pro-op, there was no statistical difference in the mean of the recursive parameters of BSs, but there was a statistical difference in the standard deviation of these recursive parameters.

**Table 3:** Statistical analysis results of CVs between Pro-op and 3 h-Pro-op

CVs	Pro-op (*n* = 18)	3 h-Pro-op (*n* = 18)	*p* values
Num_bs	7.0 (11.5)	20 (24)	0.015**
Sum_bs	1,417,106 (2,695,398)	4,164,854 (7,324,520)	0.036**
Sum_Duration_bs	39,630 (38,007)	83,282 (53,044)	0.015*
Mean_Duration	0.530 (0.300)	0.440 (0.236)	0.249**
Std_Duration	0.328 (0.342)	0.480 (0.366)	0.232*
Mean_Mag_bs	51.892 (41.220)	58.564 (33.982)	0.618*
Std_Mag_bs	10.417 (43.974)	35.874 (62.101)	0.232**
Mean_RR	0.078 (0.027)	0.103 (0.044)	0.170**
Std_RR-	0.029 (0.044)	0.069 (0.037)	0.036*
Mean_Lmean	28.048 (15.141)	30.685 (8.675)	0.618*
Std_Lmean	5.322 (8.423)	12.214 (9.214)	0.036**
Mean_ENTR	4.001 (0.523)	3.972 (0.264)	0.146**
Std_ENTR	0.191 (0.154)	0.393 (0.100)	0.015*
Mean_TT	30.794 (19.410)	33.285 (12.157)	0.646*
Std_TT	6.223 (9.759)	16.801 (12.988)	0.036**

*Pro-op*, after operation, *3* *h-Pro-op*, 3 h after operation

* Values of this line are presented as mean (standard deviation), and the statistical method is the *paired t test* when the data are normally distributed

** Values of this line are presented as median (quartile range), and the statistical method is the *rank*-*sum* test when the data are not normally distributed. CVs, characteristic values

Table 4 shows the results of the statistical analysis of CVs of Pre-op and 3 h-Pro-op. The results showed that there was no statistical difference (*p *> 0.05) among all the parameters, indicating that there was no statistical difference in the intestinal peristalsis of 3 h-Pro-op comparing with Pre-op.

**Table 4:** Statistical analysis results of characteristic values between Pro-op and 3 h-Pro-op

CVs	Pre-op (*n* = 18)	3 h-Pro-op (*n* = 18)	*p* values
Num_bs	23.5 (18.25)	20 (24)	0.979**
Sum_bs	6,150,012 (4,826,065)	5,483,036 (5,221,266)	0.979*
Sum_Duration_bs	100,070 (43,353)	83,282 (53,044)	0.865*
Mean_Duration	0.565 (0.492)	0.440 (0.236)	0.645**
Std_Duration	0.754 (0.669)	0.480 (0.366)	0.865*
Mean_Mag_bs	57.345 (42.065)	58.564 (33.982)	0.979*
Std_Mag_bs	34.746 (20.342)	42.825 (31.226)	0.865*
Mean_RR	0.114 (0.049)	0.120 (0.054)	0.979*
Std_RR-	0.050 (0.057)	0.068 (0.032)	0.865**
Mean_Lmean	29.129 (7.657)	30.685 (8.675)	0.979*
Std_Lmean	12.679 (9.011)	13.601 (7.473)	0.979*
Mean_ENTR	4.023 (0.199)	4.022 (0.237)	0.988*
Std_ENTR	0.352 (0.107)	0.393 (0.100)	0.865*
Mean_TT	32.337 (10.111)	33.285 (12.157)	0.979*
Std_TT	17.746 (11.587)	18.331 (9.279)	0.979*

*Pro-op* after operation, *3* *h-Pro-op* 3 h after operation

* Values of this line are presented as mean (standard deviation), and the statistical method is the *paired t test* when the data are normally distributed

** Values of this line are presented as median (quartile range), and the statistical method is the *rank*-*sum* test when the data are not normally distributed. CVs, characteristic values

## Discussion

General anesthesia can inhibit the gastrointestinal function of patients which includes delayed gastric emptying, small bowel transit and colonic transit [11, 12]. For this reason, patients undergoing general anesthesia cannot take food immediately after surgery. A good clinical indication of the return of coordinated bowel motility after surgery can not only guide postoperative feeding times, but also evaluate the recovery from anesthesia. In this study, the data of 5-min BSs at Pre-op, Pro-op, and 3 h-Pro-op, which reflected bowel function at each time point, were tested. After processing and analyzing the BSs data, the CVs were extracted and statistically analyzed to evaluate the changes of the BSs before, immediately after and 3 h after general anesthesia. The data of Pre-op and Pro-op were compared to see whether the intestinal function was weakened to illustrate the inhibitory effect of general anesthesia on intestinal function. The data collected at Pro-op and 3 h-Pro-op were compared to observe whether intestinal function was stronger to indicate the recovery status 3 h after general anesthesia. We also compared 3 h-Pro-op and Pre-op data to see if bowel function returned to the same state before the general anesthesia.

The statistical results of the CVs show that (1) the differences in BSs between Pre-op and Pro-op were statistically significant. (2) The differences in BSs between Pro-op and 3 h-Pro-op were statistically significant. (3) There were no significant differences between Pre-op and 3 h-Pro-op BSs. Specifically, the effect of general anesthesia on bowel function is holistic. In the statistical analysis between Pre-op and Pro-op, as well as between Pro-op and 3 h-Pro-op, there was no statistical difference in characteristic values of local BSs in the linear time-domain, but the differences in the overall occurrence frequency, total energy, and duration of intestinal sounds were statistically significant. And there was a weaker trend after surgery compared with that before surgery, and there was a stronger trend at recovery after 3 h compared with that immediately after surgery. Among the nonlinear dynamic parameters, there was no statistical difference in the mean value of the parameters that could express the complexity and stability of the local BSs, but the difference in the standard deviations of the nonlinear parameters was statistically significant, indicating that the complexity and stability dispersion degree of the BSs changed after general anesthesia. The degree of dispersion was smaller immediately after the operation, and recovered within 3 h. The statistical analysis of Pre-op and 3 h-Pro-op data showed that there were no statistical differences in either local characteristic parameters or overall characteristic parameters, whether it was linear time-domain parameters or nonlinear dynamic parameters, indicating that intestinal function had returned to the preoperative state to a certain extent 3 h after surgery.

In this study, 5 min of BSs at Pre-op, Pro-op and 3 h-Pro-op were collected to represent the intestinal status at the three periods, which has certain limitations. Under ideal conditions, the BSs should be measured continuously from the preoperative to the postoperative period, but the current surgical environment and perioperative nursing procedures do not allow for full-time measurement. However, the 5-min BSs can effectively reflect the intestinal function to a certain extent [13, 14], so research based on the 5-min BSs is effective.

Another limitation is that many empirical thresholds are used in the analysis, especially in the recognition of EBSs. Will the subjectivity of these empirical values affect the judgment of the results of general anesthesia on the change of bowel sounds? The answer is no. In this study, the same recognition threshold and methods were used to analyze and identify the intestinal sound data of the tested patients at Pre-op, Pro-op and 3 h-Pro-op, in order to judge the changing trend at these three time points, so it is not affected. Furthermore, in the current study of BSs, there is no recognized gold standard for the recognition accuracy of EBSs, and there is no standardized database to verify the accuracy. Most of the reference standards in the current study are based on the subjective judgment of clinicians, but the accuracy of such subjective judgment is also questionable [8]. Future research might use the device we made to test BSs at different intervals postoperation to determine the time it takes for intestinal function to return to pre-op status in individuals to more accurately judge the optimal postoperation feeding times.

## Conclusion

In conclusion, the hypothesis to be tested was supported as BSs changed before, immediately after and 3 h after general anesthesia in the way we predicted. The BSs weakened during surgery, and 3 h later, the BSs returned to the preoperative state. Therefore, the BSs using the parameters we identified can be used as an indicator of intestinal function changes after general anesthesia, so as to provide guidance for postoperative feeding, which is of great clinical significance.

## Methods

### Data collection

Patients’ BSs were collected using a self-developed wearable bowel sound device. The device uses a Knowles’ SiSonic MEMS microphone (SPU1410LR5H-QB), which has an ultra-wide band (UWB) flat frequency response (± 2 dB, 10 Hz–10 kHz) and a tightly matched sensitivity of ± 3 dB. Since the frequency of BSs is mainly distributed within the 100 Hz–1 kHz band, this microphone is practical for the pick-up of BSs. The bowel sound and the ambient noise acquired by the microphones are filtered and amplified through the second-order active low-pass filter first. The cut-off frequency of the low-pass filter is 2 kHz, and the magnification is 2 times. After the filter, a stage of amplification was carried out, and the amplification factor was 30. The amplified analog signal enters the analog–digital converter (12bit) of STM32L151 to realize analog-to-digital conversion. The sample rate of BSs was 8 kHz and the converted data are stored in the Micro-SD card.

### Signal processing

In the process of BSs’ acquisition, the ambient noise is easily introduced, which directly affects the quality of the BS signal. Therefore, it is necessary to remove the ambient noise to better analyze and identify the BSs. We used the noise acquisition channel of the recorder to collect the ambient noise, and the adaptive noise cancelation was used to remove the noise. Specifically, the least mean square (LMS) [15] algorithm was adopted because the LMS algorithm is more robust than the recursive least squares (RLS) algorithm [16]. The order of the filter was determined to be 32, and the step size factor was set as 0.000001 to achieve a good adaptive cancelation.

Adaptive filtering can eliminate the environmental noise, but the high-frequency noise in the signal still affects the identification and analysis of effective bowel sounds (EBSs). As an effective and practical method, wavelet denoising has achieved good results in signal and image denoising, and has been widely used in engineering applications including the enhancement of bowel sounds [17]. Donoho [18] and Walker [19] proposed a wavelet threshold denoising method. The wavelet coefficient of signal contains important information after wavelet transformation using the *Mallat* algorithm. The wavelet coefficient of the noise is less than the wavelet coefficient of the signal. By selecting a suitable threshold, the wavelet coefficients greater than the threshold are considered to be generated by BS signals and should be retained, while those less than the threshold are considered to be generated by external noise and set to zero to achieve the purpose of denoising. In the process of wavelet decomposition, the wavelet basis, the number of decomposition layers and the threshold should be determined. For the selection of wavelet basis, we chose *sym8* wavelet basis which is from the two common wavelet bases of *db* wavelet system and *sym* wavelet system. For determining the number of decomposition layers, too large or too small will both affect the final denoising effect. In this paper, the number of decomposition layers was determined to be 5 after comparing the denoising effects of different decomposition layers. For the determination of threshold value, the *Birge*-*Massart* [20] algorithm was used to obtain the threshold value of each layer of one-dimensional wavelet transform, and soft threshold function was used for denoising. For bowel sound signals, there is no standard signal to refer to, so the wavelet denoising was used combining with adaptive filtering, to keep the frequency response range below 1 kHz [21] which is the main frequency range of bowel sounds.

**Fig. 1:**
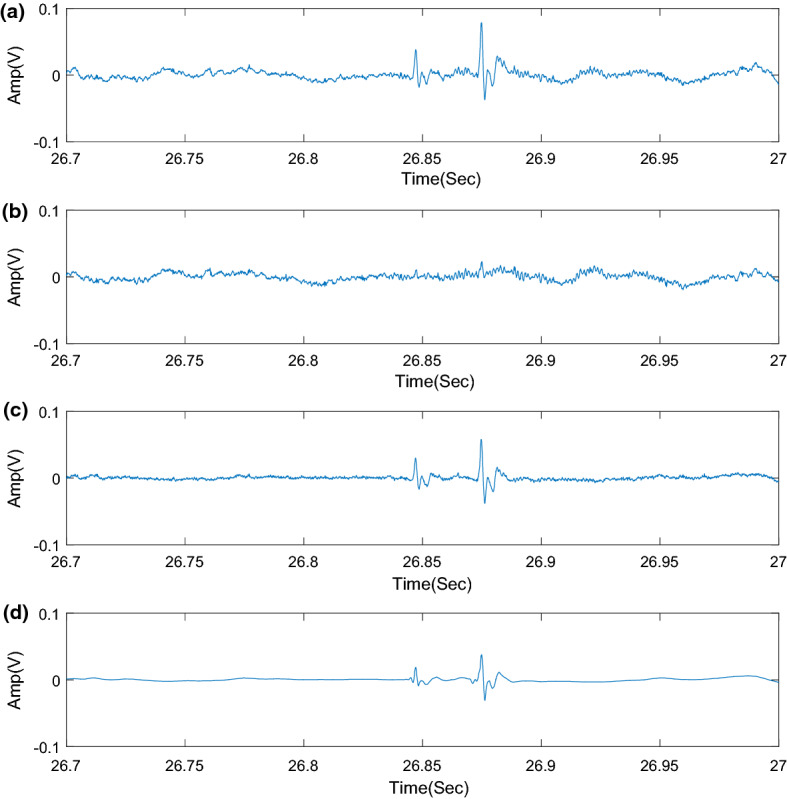
The bowel sounds’ signal after adaptive filtering and wavelet denoising. **a** The original signal is obtained from the microphone after amplification without any cancelation, and **b** the ambient noise is collected from the other microphone. **c** The signal after LMS adaptive filter. **d** The signal after wavelet denoising

After the adaptive filtering and wavelet denoising, the waveform (Fig. 1) can be used to identify EBSs. The fractal dimension (FD) can quantitatively describe the complexity of the signal. The FD of EBSs is different from that of background sounds [22]. To calculate the FD of a time series, we can either reconstruct the phase space first and then calculate the correlation dimension of the time series [23–25] or directly calculate the FD in the time-domain. The time series in this paper was the audio signal with a high sampling rate and large data volume, so the FD was calculated directly in the time-domain. The Katz method [22, 26] used in FD calculation can effectively judge the randomness of waveforms. When calculating the FD of the BS signal, we employed a sliding window to realize the short-time processing of audio signals. The length of the sliding window was set to *int* (0.006*fs), where *int* indicates the integer part of the argument, and *fs* is the sampling frequency of the BS signal. The constant 0.006 is empirically set and justified by the efficient performance of the algorithm [22]. The FD of the data in the sliding window is calculated. To ensure that the length of the data before and after calculating the FD is equal, the first and the last FD are used to make up the data at both ends. After the FD sequence is calculated, the peak value is extracted to ensure the effective recognition of the BSs. The peak extraction method adopts the FD-peak peeling algorithm (FD-PPA) [22].

FD-PPA makes the EBSs more obvious in the waveform, but the voice endpoint detection (VAD) technology is needed to extract the EBSs. The purpose of VAD technology is to identify the starting point and ending point of EBSs accurately from a segment of the signal containing EBSs to distinguish the EBS and the non-BS signal. It is an important aspect of speech processing technology. As for the BS signal, we identified the EBSs which satisfied certain conditions, while the others are considered as non-BS signals. In this paper, the time series after FD-PPA were used as the input sequence to judge the starting and ending points of EBSs. The threshold for entering the BS segment, the length threshold of identified noise, and the maximum allowed mute length in the BS segment are set. Based on the above three thresholds, the endpoint of EBSs was determined. As a rule of thumb, the first is the threshold for entering BS segments which was set to 1.01. When the input value is greater than 1.01, it is considered to be the starting point of EBSs. The second parameter is the minimum duration threshold of the EBS signal, and the BS segment less than this threshold is considered as noise. And this threshold is set to 50 ms. The maximum mute length allowed in the BS segment is the third threshold which was set to 250 ms. If the mute length in the BS segment is less than this value, the BS is considered unfinished; otherwise, the BS segment is considered finished.

After the VAD, there are also many kinds of vocal signals mixed in, such as heart sounds, breath sounds and background noises similar to BSs. Limited to the problem of environmental noise collection and filter residue, we set three thresholds to remove three kinds of residual noise based on experience. Specifically, the envelope of each EBS was obtained by complex analytic wavelet transformation [27]. Then, we excluded the sound segment whose envelope maximum value was less than 0.037 V, which meant that a sound segment with a too small amplitude is considered as noise. In the measured data, the confounding heart sounds is obvious. We extracted the envelope of sound segment and calculated the peak number. And based on the experience in judging heart sounds we ruled out the sound segment whose peak value was less than 3. We also found that for BS segments with a very small signal-to-noise ratio, there was residual noise and it was identified as a gut sound, which also needed to be removed. As for this speech segment with residual noise, we filtered out the envelope peak number which was more than 3 in the length of 1000 sampling points based on experience.

### Characteristic values’ extraction

The characteristic values (CVs) can quantitatively reflect the characteristics of BSs, so we extracted linear and nonlinear CVs for quantitative evaluation and statistical analysis. The linear CVs are mainly time-domain parameters, as shown in Table 5.

**Table 5:** The linear CVs of EBSs

Linear CVs	Calculation methods	Physiological significance
Num_bs	The number of identified effective bowel sounds during the measurement	Frequency of bowel sounds in the 5 min
Sum_bs	The sum of the absolute values of the identified effective bowel sounds	Reflecting the total energy of the bowel sounds
Sum_Duration_bs	The sum of the duration of the identified effective bowel sounds	Reflecting the total duration of bowel sounds
Mean_Duration	The mean of the duration of effective bowel sounds	Mean duration of effective bowel sounds
Std_Duration	The standard deviation of the duration of effective bowel sounds	Standard deviation of duration of effective bowel sounds
Mean_Mag_bs	The mean of the mean absolute value of effective bowel sounds	The average energy of effective bowel sounds
Std_Mag_bs	The standard deviation of the mean absolute value of effective bowel sounds	The standard deviation of the energy of the effective bowel sound

*CVs* characteristic values, *EBSs* effective bowel sounds

Physiological signals have been shown to be chaotic [28]. As the basic physiological signal, gut sound also has nonlinear dynamic characteristics. Therefore, nonlinear CVs were calculated in this paper. Recurrence quantification analysis (RQA) [29] can measure the complexity of a short and non-stationary characteristic signal with noise [30]. It has been broadly applied in the analysis of physiological data [31–33]. In this paper, phase space reconstruction was carried out for each EBS signal. Based on the recursive graph, recursive quantitative analysis was carried out and quantitative parameters were extracted [34], as shown in Table 6. There are multiple EBSs in each period, so to realize the subsequent statistical analysis, the mean value (Mean_) and standard deviation (Std_) of each CV in each period were calculated.

**Table 6:** The nonlinear CVs of EBSs

Nonlinear CVs	Calculation methods	Physiological significance
RR	The percentage of recurrent points falling within the specified radius	Reflect the similarity of signal fluctuation
Lmean	The mean of the diagonal lengths in recurrence plot	Related to the separation velocity of adjacent trajectories
ENTR	The Shannon information entropy of all diagonal line lengths	A measure of signal complexity
TT	The average length of vertical line structures	Degree of system stability

*CVs* characteristic values, *EBSs* effective bowel sounds

**Fig. 2:**
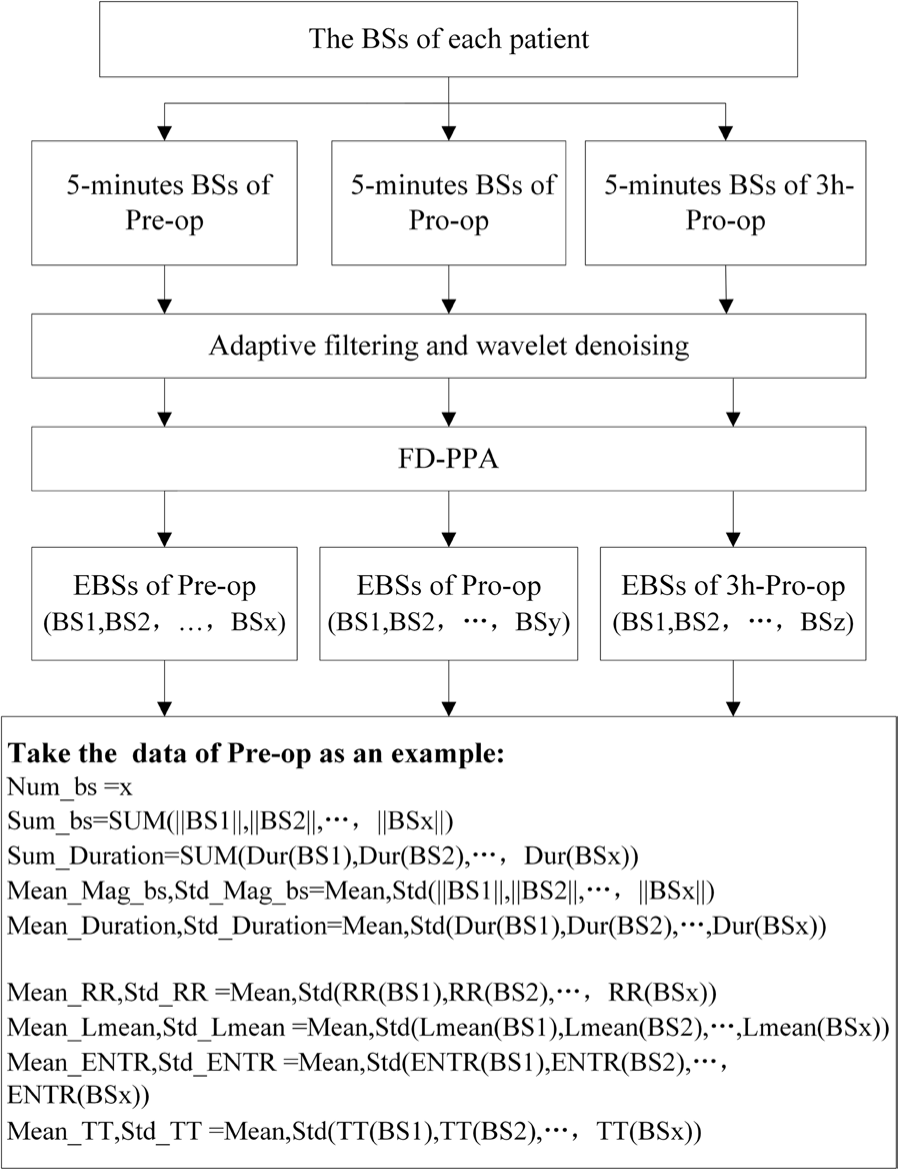
Overview of BSs data acquisition, processing, and analysis. BS is short for the bowel sound. Pre-op is short for before operation. Pro-op is short for after operation. 3 h-Pro-op is short for 3 h after operation. FD-PPA is short for FD-peak peeling algorithm. EBS is short for the effective bowel sound

Figure 2 shows an overview of BSs data acquisition, processing, and analysis, and $f\left( t \right)$ is calculated as Eq. (1).

1 $$ f\left( t \right) = \sqrt {\mathop \smallint \nolimits_{ - \infty }^{\infty } \left| {f\left( t \right)} \right|^{2} dt} $$

### Statistical analysis

We attempted to analyze the differences in BSs at Pre-op, Pro-op and 3 h-Pro-op. The CVs can quantitatively represent the signals, so we conducted statistical analysis on the CVs of the 26 patients between Pre-op and Pro-op, the 18 patients between Pro-op and 3 h-Pro-op, and the 18 patients between Pre-op and 3 h-Pro-op. And then determined whether there were statistical differences. The CVs for statistical analysis included linear CVs and the means and standard deviations of nonlinear CVs.

Statistical analyses were performed using IBM SPSS Statistics 25. Each set of statistical analyses was taken from the same patients at two moments. And the normal distribution test was performed before statistical analysis. Normality test is performed for each set of data using Shapiro–Wilk normal test. If the data are normally distributed, the significance level (i.e., *p* value) should be greater than 0.05; otherwise, the *p* value should be less than 0.05. So for data satisfying normal distribution, the parametric statistical method of paired *t* test was used. Otherwise, the *rank*-*sum* test was used. A value of *p* < 0.05 was considered to indicate statistical significance.

## Data Availability

The datasets generated during and/or analyzed during the current study are available from the corresponding author at reasonable request.

## Abbreviations

BSs: Bowel sounds
Pre-op: Before operation
Pro-op: After operation
3 h-Pro-op: Three hours after operation
EBSs: Effective bowel sounds
CVs: Characteristic values
DMR: Dynamic magnetic resonance
LMS: Least mean square
FD: Fractal dimension
VAD: Voice endpoint detection
RQA: Recurrence quantification analysis
